# Detection of Structural Variants by NGS: Revealing Missing Alleles in Lysosomal Storage Diseases

**DOI:** 10.3390/biomedicines10081836

**Published:** 2022-07-29

**Authors:** Valentina La Cognata, Sebastiano Cavallaro

**Affiliations:** Institute for Biomedical Research and Innovation, National Research Council, 95126 Catania, Italy; valentina.lacognata@cnr.it

**Keywords:** lysosomal storage diseases, diagnosis, tNGS, structural variants, CNVs

## Abstract

Lysosomal storage diseases (LSDs) are a heterogeneous group of rare multisystem metabolic disorders occurring mostly in infancy and childhood, characterized by a gradual accumulation of non-degraded substrates inside the cells. Although biochemical enzymatic assays are considered the gold standard for diagnosis of symptomatic patients, genotyping is a requirement for inclusion in enzyme replacement programs and is a prerequisite for carrier tests in relatives and DNA-based prenatal diagnosis. The emerging next-generation sequencing (NGS) technologies are now offering a powerful diagnostic tool for genotyping LSDs patients by providing faster, cheaper, and higher-resolution testing options, and are allowing to unravel, in a single integrated workflow SNVs, small insertions and deletions (indels), as well as major structural variations (SVs) responsible for the pathology. Here, we summarize the current knowledge about the most recurrent and private SVs involving LSDs-related genes, review advantages and drawbacks related to the use of the NGS in the SVs detection, and discuss the challenges to bring this type of analysis in clinical diagnostics.

## 1. Introduction

Lysosomal storage diseases (LSDs) are inborn metabolic disorders caused by the absence or deficiency of one specific component in the lysosomal pathway, resulting in the accumulation of undegraded substrates in multiple organs [1]. These disorders encompass a group of about 70 different entities that can be subclassified according to the enzyme deficit and the specific involved substrate: lipid storage diseases, mucopolysaccharidoses, glycoproteinoses, lipofuscinosis, lysosomal integral membrane proteins diseases, post-translational modifications dysfunctions and lysosomal related organelles (LRO) disorders [2]. Although each disorder *per se* is rare, with estimated incidences ranging from 1 in 50,000 to 1 in 250,000 live births, they are relatively common disorders when considered collectively (1:5000 live births) [2].

The storage process leads to a broad spectrum of clinical manifestations depending on the specific substrate and site of accumulation [3]. Signs and symptoms may occur from prenatal period to adulthood, and develop progressively over time from mild to extremely severe forms, and could include organomegaly, pulmonary and cardiac problems, facial dysmorphisms, musculoskeletal abnormalities, cognitive decline, deafness, blindness and movement problems, all having a severe impact on patients’ prognosis and quality of life [1,3,4].

From a genetic point of view, LSDs are Mendelian monogenic disorders, inherited mainly in an autosomal recessive or in an X-linked manner (i.e., Fabry, Hunter, and Danon diseases), and are caused by mutations in genes encoding for acid hydrolases, integral membrane proteins, activators and transporter proteins, or other accessory proteins related to lysosomal function [2,5]. Although biochemical enzyme assays are considered the gold standard for diagnosis of symptomatic patients, genotyping is a requirement for inclusion in enzyme replacement programs and is a prerequisite for carrier tests in blood relatives and DNA-based prenatal diagnosis. Routine diagnostic DNA analysis usually focuses on genetic coding regions using PCR reactions and subsequent Sanger sequencing and is successful in identifying single nucleotide variants (SNVs) disrupting protein function [2]. Despite this, a number of patients with suspected LSDs have other types of genomic variations and do not achieve a definitive clinical diagnosis for many years. The current advancement in next generation sequencing (NGS) technologies is now providing a powerful diagnostic tool for patients affected by LSDs who escape the diagnosis by traditional genetic investigation, and is offering a range of new opportunities for genomic medicine [6,7]. Thanks to these high-throughput sequencing techniques, geneticists and clinicians can go beyond the search for single nucleotide variants (SNVs) hotspot mutations in the coding sequence of responsible genes, and more easily identify small insertions and deletions (indels) and major structural variations (SVs) responsible for the pathology [8,9,10].

SVs, in particular, include genetic variants greater than 50 base pairs (bp) in length and comprise chromosomal inversions, insertions, translocations, further complex rearrangements and genomic imbalances (duplication or deletions) commonly referred to as copy number variants (CNVs) [11]. Improvement in SV detection is enhancing our understanding of the intricate interplay between genetic makeup and associated phenotype, unraveling their contribution in disease etiology, gene expression regulation and phenotypic diversity in rare diseases, including LSDs. During the last years, great progress has been made in order to standardize the detection of SVs with sufficient accuracy and precision for use in clinical diagnostics, but the challenge still remains open [11].

Here we summarize the current knowledge about the most recurrent and private genomic SVs involving LSDs-related genes, review advantages and drawbacks related to the use of NGS in the SVs detection, and discuss the challenges to bring this type of analysis in clinical diagnostics.

## 2. CNVs Are the Major SVs

Copy number variants (CNVs), encompassing losses or gains of genomic DNA segments larger than 50 bp, are the most common type of SVs in the human genome and represent a major source of human genetic variation accounting for disease and population diversity [11,12]. Recent studies revealed that de novo locus-specific mutation rates appear much higher for CNVs than for SNVs [13,14,15], and that the frequency of a CNV shows strong anticorrelation with its size and gene density [16].

Although several genomic copy number imbalances are presumably benign, their role in the pathogenesis of various diseases has been recently gaining increasing attention. Benign CNVs are frequently small, intergenic, or comprise genes that can tolerate copy number changes, while pathogenic CNVs are significantly enriched in dosage sensitive-genes or in regions with constrained evolutionary patterns of gene duplication and loss [17]. In the early era of CNV detection, large CNVs (>500 kb) appeared to be associated with genomic disorders only. However, it is now clear that these types of alterations may cause or influence Mendelian diseases as well complex traits [12,18].

Disease-causing genomic rearrangements can be either recurrent or non-recurrent. The main fraction of recurrent CNVs arises from nonallelic homologous recombination (NAHR) mechanisms mediated by high-identity segmental duplications or Alu repeats [13,19]. These genomic disorders are highly penetrant, can occur in different haplotype backgrounds in multiple unrelated individuals in a relatively short period of time, and are under strong negative selection. CNVs can also arise from non-recurrent mechanisms, which include non-homologous end-joining (NHEJ), Fork Stalling and Template Switching (FoSTeS), and microhomology/microsatellite-mediated break-induced repair. These rearrangements do occur at a relatively lower frequency and assessing their clinical relevance is problematic, since it requires the screening of many thousands of patients to establish pathogenicity [13,19].

The molecular mechanisms by which a CNV causes abnormal phenotypes include gene-dosage sensitivity, gene interruption, gene fusion at the breakpoint junctions, deletion of a regulatory element, the unmasking of a recessive allele or a functional polymorphism [20], and alteration of non-coding regulatory elements (promoters or enhancers) [18]. The advances in whole-genome technologies are enabling the discovery and characterization of large and intermediate SVs, but the landscape of CNVs still remains largely unexplored, especially for those embedded within complex regions of the human genome.

## 3. SVs in LSDs

More than 65 genes responsible for the wide spectrum of LSDs have been described so far [1,2]. For most of these conditions, clinically relevant homozygous or compound heterozygous SVs (either recurrent or private) have been described in LSD patients in single case reports or in cohort studies and are collected in international reference databases (e.g., ClinVar, Decipher, DGV, GnomAD), where the reader is referred to for a complete and comprehensive consultation. Here, to the best of our knowledge, we will briefly report both past and recently identified SVs altering LSDs-related genes (Supplementary Table S1), although the creation of an extensive repository is far beyond the scope of the present work. For simplicity, pathologies will be discussed separately in subgroups.

### 3.1. Enzyme Deficiency Disorders

#### 3.1.1. Lipid Storage Diseases

Lipid storage diseases include both the large group of sphingolipidoses and Wolman disease, which is caused by lysosomal acid lipase deficiency.

Sphingolipidoses are characterized by abnormal storage of various phospholipids with a sphingosine group, and include: Fabry (*GLA*), Niemann–Pick A/B (*SMPD1*), Krabbe (*GALC*), Gaucher (*GBA*), Tay–Sachs (*HEXA*), Farber (*ASAH1*), Sandhoff (*HEXB*) and Metachromatic Leukodystrophy (*ARSA*) [2]. A number of disease-causing gene CNVs have been described, including: (i) a gross deletion involving *ASAH1* (g.728_18197del (c.126-3941_382 + 1358del) in a child with severe Farber disease [21]; (ii) a whole-gene deletion of *ARSA* in a patient with infantile Metachromatic Leukodystrophy [22]; (iii) two single-exon deletion involving *GALC* exon 12 and 14 and multiple contiguous exons loss (exons 11–17) in patients with Krabbe disease [23]; (iv) a recurrent 5′-end 16 kb deletion comprising *HEXB* promoter region, exons 1–5 and part of intron 5 in Sandhoff disease patients [24,25], and further partial deletions comprising intron 1-exon 2 [25] and exons 1–5 [26]; (v) multiple complex pathogenic duplications or deletions of *SMPD1* in patients with acid sphingomyelinase deficiency [27].

Two genes of this subgroup (*GBA* and *GLA*) undergo frequent SVs, as better discussed here below.

The *GBA* gene is located in an unstable chromosomal region, structurally subject to misalignments, reciprocal/nonreciprocal homologous recombination events and DNA rearrangements (deletions, duplications, inversions and fusions), representing the main cause of pitfalls in Gaucher disease mutational analysis [9,28]. This gene-rich locus encompasses seven genes, *Alu* sequences and a highly homologous 5.7-kb pseudogene (*GBAP1*) located approximately 16 kb downstream *GBA* showing an exons/intron organization similar to the functional gene [9]. Total gene deletion and more than 20 recombinant alleles (including *Alu*-mediated large deletion) have been characterized to date, with RecNcil and Recdelta55 being the most common either alone or in combination with additional point mutations [9,28,29,30,31,32,33]. In particular, RecNcil derives from a cross-over junction area from intron 9 to exon 10, resulting in the incorporation of a segment of *GBAP1* that includes several missense mutations into the functional *GBA*, whereas Recdelta55 encompasses a 55-bp deletion in exon 9 of the *GBA* corresponding to the deleted portion of the pseudogene. Given this highly unstable locus, the need to adopt adequate strategies to avoid misdetection of *GBA* recombinant mutations is clear. To this end, different strategies were described to provide proper *GBA* genotyping and genetic counseling, such as specific procedures for NGS-based targeted library preparation and data analysis [9] and clinical exome sequencing in combination with MLPA assays [10].

Regarding *GLA*, among the approximately 1000 gene mutations documented in the Human Gene Mutation Database, about 30 are SVs from 0.1 to several kb of size and predominantly include gross deletions, resulting from various recombination events (e.g., short regions of homology, short inverted repeat sequences, *Alu*-*Alu* recombination, insertion of retrotransposon L1, double strand breaks repaired by a non-homologous end-joining mechanism) [34]. Southern hybridization, restriction analyses, MLPA, and multiplex PCR assays have been useful in detecting these complex rearrangements [34,35,36,37,38], which are often missed by routinary DNA sequencing (especially in female Fabry disease patients). Recently, the use of NGS-based read amplicon technology proved successful in resolving a complex case [39].

Wolman’s disease (WD) is a rare and fatal infantile metabolic disorder caused by lysosomal acid lipase deficiency. Very recently, a 4-month-old infant presenting with hepatosplenomegaly, failure to thrive and other abnormalities carrying a homozygous deletion of exons 9–10 of the in *LIPA* gene has been described [40].

#### 3.1.2. Mucopolysaccharidoses

Mucopolysaccharidoses (MPS) are a group of inherited LSDs characterized by accumulation of undegraded glycosaminoglycans (GAGs or mucopolysaccharides) in the lysosomes of cells, resulting in severe symptoms including coarse facial features, cognitive retardation, hepatosplenomegaly, hernias, kyphoscoliosis and corneal clouding. The occurrence of SVs is frequent in this group of pathologies, although often they are undetected and result in failed diagnosis. Indeed, several MPS-related genes undergo recurrent or private SVs as described below. Particular emphasis will be given to *IDS*, the causative gene of MPS II [41].

CNVs in MPS-related genes have been characterized and encompass: (i) deletion of exon 14-3′UTR, and duplication of exon 2-intron 12 in *IDUA* in patients affected by MPS I [42,43]; (ii) deletion of *SGSH* exons 1–5 in MPS IIIA patients [44]; (iii) *Alu*-mediated deletion of *NAGLU* exons 3–4 in patients with MPS IIIB/Sanfilippo type B syndrome [45,46]; (iv) heterozygous deletion of exon 15 [47] and homozygous deletion of exons 9–10 in *HGSNAT* in MPS IIIC or Sanfilippo type C patients [43]; (v) deletions of *GNS* exon 1, 2–3, 6–7, 9–14 in patients affected by MPS IIID/Sanfilippo disease type D [48,49]; (vi) deletion of multiple contiguous exons including 1–3, 2–4, 2–5, 3–14, 5–8, 9–14, 10–14, 11–12, and single exon 5 and 13 of *GALNS* in patients with MPS IVA/Morquio A [43,50,51,52,53]; (vii) *ARSB* deletion of exons 2–3, exon 4 and exon 5 MPS VI patients [54,55,56].

*IDS* deserves a separate discussion, which is responsible for MPS II or Hunter syndrome. The mutational spectrum associated with this gene is quite heterogeneous, ranging from point mutations to recurrent gross genomic rearrangements, occurring approximately in 10–20% of MPS II patients [41]. *IDS* spans about 24 kb in Xq28 and consists of nine exons. A pseudogene (*IDSP1*—also known as *IDS2*), located telomeric to the functional *IDS* gene, contains sequences homologous to exon 2, intron 2, exon 3 and a chimerical fragment of intron 3–intron 7, making this region prone to the occurrence of non-allelic homologous recombination events [41]. Indeed, whole *IDS* gene deletion (with or without the involvement of further neighboring genes) and partial *IDS* exon deletions have been reported in multiple studies, mainly in patients with a severe Hunter syndrome presentation [41,57,58,59,60,61,62,63,64,65,66,67,68,69,70,71]. Furthermore, *IDS* exons duplications, *IDS1/IDS2* inversions, chimeric *IDS-IDS2* allele and other large complex rearrangements have been described [41,57,58,59,60,61,62,63,64,65,66,67,68,69,70,71], as well as deletions in the promoter region in patients with a mild phenotype [72]. Recently, a case report described a girl with comorbidity of Hunter and Turner syndromes caused by a partial deletion in the long arm of chromosome X of paternal origin and a deletion of *IDS* inherited from the mother [73].

#### 3.1.3. Glycogen Storage Disease

Genetic variants in *GAA* are responsible for Pompe disease (PD) or glycogen storage disease type II, a metabolic myopathy with a wide spectrum of clinical presentation caused by acid a-glucosidase enzyme deficiency. Depending on the residual *GAA* enzyme activity, the disease either develops during the first months of life as the classic infantile Pompe disease (IOPD), or later in life (childhood, adolescence or adulthood) with a milder phenotype known as late-onset Pompe disease (LOPD) [74]. Enzyme replacement therapy (ERT) should be started before symptoms appear in order to achieve optimal outcomes, thus highlighting the importance of detecting PD patients in the very early stage, and encouraging the development of newborn screening studies also with the help of NGS applications [75,76]. Multiple CNVs affecting this gene have been identified, including the most common deletion of exon 18 [77,78,79,80], and the deletion of exons 2–3 [81], exons 2–4 [80,82,83], introns 7–15 [80,84], introns 17–18 and exons 15–20 [80].

#### 3.1.4. Glycoproteinoses

Glycoproteinoses are among the rarest and less studied LSDs and include aspartylglucosaminuria (*AGA*), fucosidosis (*FUCA1*), galactosialidosis, (CTSA), α-mannosidosis (*MAN2B1*), β-mannosidosis (*MANBA*), Schindler disease (*NAGA*), and sialidosis (*NEU1*). Being rare diseases, their genetic/genomic characterization is less detailed. Nonetheless, two large deletion (encompassing exon 4, and exons 7/8) and a 66bp duplication in exon 6 have been reported in homozygous state in *FUCA1* in a patient with fucosidosis [85]. More recently, a deletion of *NEU1* exon 2 was reported in a patient with type 1 sialidosis [86], as well as a heterozygous 27.5 kb deletion involving the whole coding exons of *NEU1* in a girl with reduced visual acuity [87]. The power of NGS in the identification and characterization of SVs have been proved also in a case report describing a 15-year-old girl with β-Mannosidosis, revealing a complex homozygous rearrangement characterized by a partial intragenic inverted duplication of *MANBA*, and resulted in the mRNA skipping of multiple contiguous exons [88].

### 3.2. Disorders of Post-Translational Modification

Disorders of post-translational modification include multiple sulfatase deficiency and mucolipidoses (ML) II and III, and result from mutations in genes that have a role in biochemically modifying lysosomal hydrolases. In particular, mutations in *GNPTAB* cause both the severe type of ML (ML II alpha/beta) and the attenuated type of ML (ML III alpha/beta, or Pseudo-Hurler polydystrophy) [89]. Some genomic rearrangements altering *GNPTAB* structure have been described and include a duplication of exon 2 in both ML II and ML III patients [89], an *Alu*–*Alu*-mediated large homozygous genomic deletion (897 bp) encompassing *GNPTAB* exon 19 in a ML II a/b patient [90], and a deletion of *GNPTAB* exon 9 in a Chinese patient in combination with a point mutation [91].

### 3.3. Disorders of Integral Membrane Proteins

Disorders of integral membrane proteins include six different LSDs that, with the exception of cystinosis and Niemann–Pick type C, are rare in the general population. For five of them, both recurrent and private SVs have been reported in multiple studies.

The most common mutation causing cystinosis is a 57-kb deletion on human chromosome 17p13 that removes the majority of *CTNS* coding region, as well as some further adjacent genes [92]. This deletion occurs in ~60% of United States and Northern Europe patients [93,94]. A set of additional smaller SVs (from hundreds of base pairs to tens of kb) have been described in single studies (i.e., 13 kb deletion [95]; 266 bp duplication [96], deletion of exons 4–5 [97]; 10 kb deletion [98]; >1.7 kb deletion [99]).

Niemann–Pick type C is caused by mutations in either genes *NPC1* (95%) or *NPC2* (5%). More than 300 disease-causing mutations have been identified so far in both genes, including indel, missense, nonsense and splicing mutations [100]. Although very rare, SVs have been documented in research literature, providing insights into missing *NPC1/2* mutant alleles. In particular, *NPC1* whole-gene deletion has been characterized in Niemann–Pick type C patients [101,102], as well as two different larger structural variants encompassing *NPC1* and flanking genes (*RMC1* and part of *ANKRD29* in the first one, *ANKRD29* and *LAMA3* in the second one) [100], and a homozygous deletion of exons 2 and 3 of *NPC2* [103].

Similarly, a partial *MCOLN1* gene deletion (c.1_788del) has been detected and described in patients with Mucolipidosis Type IV and identified as founder mutation in the Ashkenazi Jewish population [104], while a homozygous 94 bp deletion and a homozygous deletion of exons 8–9 in *SLC17A5* were found in a child and in a prenatal hydrops fetalis with infantile free sialic acid storage disease, respectively [105,106].

Of particular interest is the Xq24 chromosomal region harboring *LAMP2*, whose mutations cause Danon disease. Several large deletions that alter the *LAMP2* exon copy number have been described in male patients with Danon disease and involve repetitive sequence motifs, such as *Alu*-mediated or TA-rich repeat elements [107]. Recent detailed investigations highlighted a spectrum of peculiar SVs in female patients, such as heterozygous multi-exon *LAMP2* deletions and de-novo *Alu*-mediated Xq24 rearrangement deleting the distal part of *CUL4B* and the complete sequences of *LAMP2*, *ATP1B4*, *TMEM255A* and *ZBTB33* genes [108,109]. In addition, a tandem and likely *Alu*-mediated duplication of exons 4–5 in the *LAMP2* gene has been identified in two brothers with typical Danon disease phenotype and was mosaically distributed in the somatic tissues of their clinically asymptomatic mother [110].

### 3.4. Neuronal Ceroid-Lipofuscinoses

The neuronal ceroid-lipofuscinoses are a group of neurodegenerative disorders, mostly of childhood onset, characterized by progressive neuroretinal symptomatology, progressive accumulation of auto-fluorescing waxy lipopigments (ceroid-lipofuscin) within the brain, cerebral atrophy, epilepsy, dementia and early death. These disorders were initially classified on the basis of age at onset and consisted of four major groups: infantile, late infantile, juvenile and adult. More recently, the classification was updated to include the genetic causative gene as well as the age of onset [1]. Currently, a total of 14 genes have been identified as the monogenic causes of NCLs [2].

Ceroid lipofuscinosis, neuronal type 3, or Batten disease, is the most prevalent form and is associated with biallelic mutations in *CLN3*, characterized by a common founder 1.02-kb intragenic deletion mutation (removing exons 7 and 8) occurring in homozygosity in 76% of patients, and compound heterozygosity in an additional 22% of patients [111,112].

Intragenic deletions encompassing exon 4 and a large pathogenic deletion at 13q21.33-q31.1 were described for *CLN5*, whose mutations are responsible of a late-infantile form of NCLs, originally described in the Finnish and Northern European populations [113,114].

Single allele deletions that complicate genetic diagnosis have also been reported for *CLN8*. The first de novo terminal deletion of the short arm of chromosome 8p23.3 was described in a Irish patient [115], followed by a Turkish family carrying a 2.6 kb *CLN8* intragenic deletion [116]. More recently, three unrelated patients (from Argentina and Britain), each carrying large deletions encompassing the 37 kb *CLN8* gene, were characterized by microarray analysis [117].

### 3.5. Disorders of LROs

The most notable LRO disorders include the multiple variants of Hermansky–Pudlak (HPS) disease, the Griscelli and the Chédiak–Higashi syndromes, which are all characterized by hypopigmentation (owing to a melanosome defect) and prolonged bleeding (owing to a platelet δ granule defect). Of the nine HPS subtypes, the first one was identified in a genetic isolate of central Puerto Rico and is associated to mutations in *HPS1* gene [118]. A single hemizygous patient carrying a large deletion (13,966-bp) involving *HPS1* and an adjacent gene, *C10ORF33*, has been reported to date [119].

The gene responsible for Hermansky–Pudlak type 2, *AP3B1*, is located on the long arm of chromosome 5 (5q14.1) and encodes the β3A subunit of the AP-3 complex [120]. Few cases of SVs in this genomic location are described in the literature. The first one, characterized in 2006, consists of a homozygous deletion of *AP3B1* exon 15 (8168 bp), which completely abrogates the proper assembly and the stability of the AP-3 complex [121]. More recently, exon–exon analysis revealed a deletion of single exon 14 and exons 10–25 in two patients with Hermansky–Pudlak type 2 [120]. A consanguineous female infant with reduced pigmentation, neutropenia and recurrent infections was shown to carry a homozygous pericentric inversion inv(5)(p15.1q14.1) that likely disrupts *AP3B1* at the breakpoint, thus providing a novel pathogenic mechanism for this disease [122].

## 4. Advantages and Drawbacks of NGS Targeted Panel Usage for the Detection of SVs in LSD-Related Genes

NGS technologies currently represent powerful tools for the characterization of SVs in human genomes by providing faster, cheaper and higher-resolution testing options [123]. Data produced by these platforms allow both the identification of point mutations, indels and SVs in a single experimental workflow and guarantee greater coverage, higher resolution, more accurate copy number estimation and more precise breakpoint detection [123,124].

Multiple algorithms have been developed to detect SVs based on different features that can be extracted from NGS data and are based on indirect inferences, such as paired-end mapping, splits read, de novo assembly and depth of coverage (DoC), for which we refer the readers to more detailed works [125,126,127,128]. Very briefly, in paired-end sequencing, DNA fragments are assumed to have a specific insert-size distribution. Tools based on the paired-end mapping approach identify CNVs by detecting fragments with a discordance between mapped paired-reads whose distances differ significantly from the predetermined average insert size. The split read approach uses reads from pair-end sequencing in which only one read of the pair is reliably mapped and the other either partially or completely fails to map to the genome. In de novo assembly, DNA fragments are reconstructed from short reads by assembling overlapping reads, and the assembled fragments are then compared to the reference genome to identify regions with different copy numbers. Finally, the DoC approach is based on the assumption that the depth of sequence coverage at any target is generally correlated with the initial copy number of the corresponding region [125,126]. Sensitivity as well as specificity is highly dependent on the SVs detection algorithms used and the estimations of the false-negative and false positive rates greatly vary [125,126]. Unfortunately, there is currently no single informatic method able to identify the full range of structural DNA variations, and multiple complementary SVs callers are required for robust variant detection.

Despite most of the SVs callers having been developed for whole-exome sequencing (WES), recent efforts were implemented to adapt these analysis workflows into adequate strategies for custom or commercial targeted gene panels (tNGS), which offer greater mean coverage of clinically relevant genes at minor cost, and allow more accurate and sensitive detection of disease-related small CNVs encompassing one or more contiguous genes and exons [125]. In the case of LSDs-related genes, for example, specific procedures for library preparation and data analysis are currently adopted when the tNGS panel targets unstable genomic regions such as for *GBA* and *IDS* described above, characterized by highly homologous regions or locus with pseudogenes in proximity [9,125]. Indeed, these similarities complicate the alignment of reads corresponding to these regions, significantly affecting data output and must be taken into account in data analysis.

**Figure 1:**
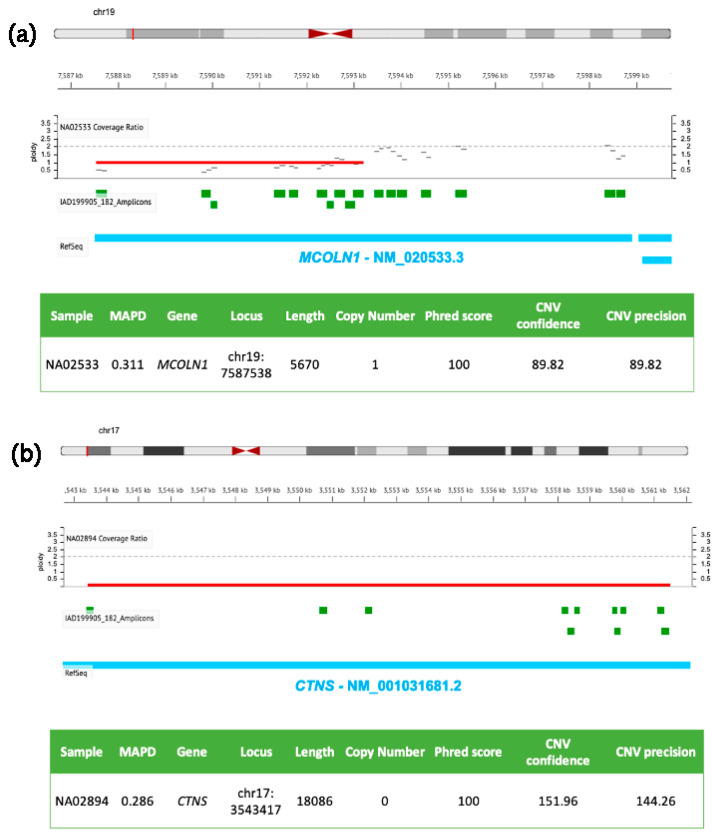
Molecular details of CNVs detected in *MCOLN1* and *CTNS* by NGS. DNA samples (NA02533 in (**a**) and NA02894 in (**b**)) were isolated from clinically diagnosed donor subjects obtained from the NIGMS Human Genetic Cell Repository at the Coriell Institute for Medical Research. Targeted NGS analysis was performed by using the tNGS panel previously described [126] and analyzed with the Ion Reporter Software (default parameters for germline CNVs, Median of the Absolute values of all Pairwise Differences < 0.4, CNV Confidence > 20, CNV precision > 10).

**Figure 2:**
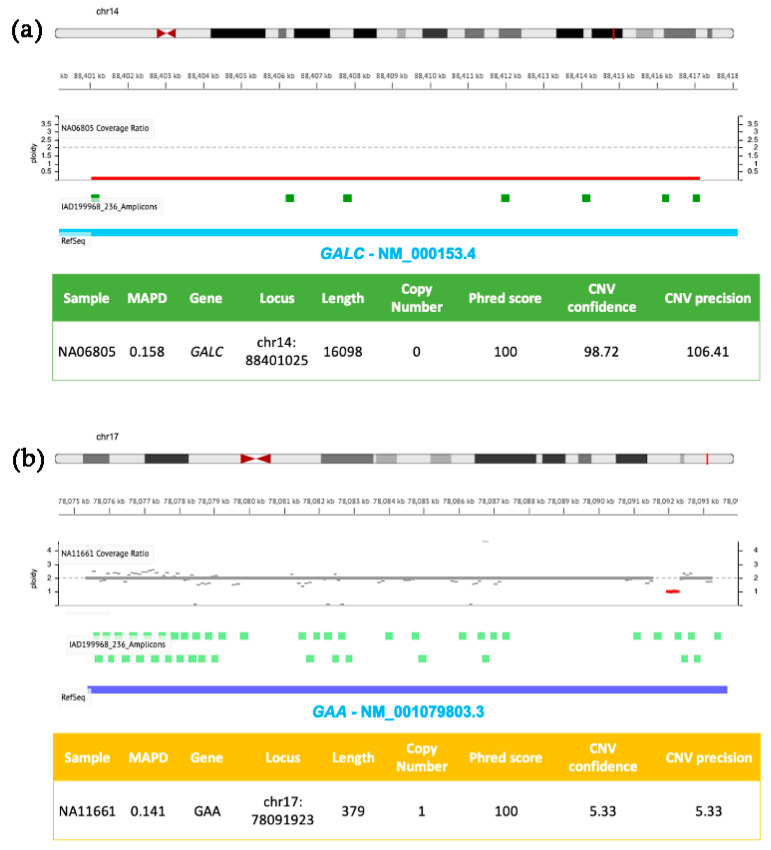
Molecular details of CNVs detected in *GALC* and *GAA* by NGS. DNA samples (NA06805 in (**a**) and NA11661 in (**b**)) were isolated from clinically diagnosed donor subjects obtained from the NIGMS Human Genetic Cell Repository at the Coriell Institute for Medical Research. Targeted NGS analysis was performed by using the tNGS panel previously described [5] and analyzed with the Ion Reporter Software (default parameters for germline CNVs were as described in Figure 1 with the exception of panel b, where CNV Confidence > 5 and CNV precision > 5).

Specific user-friendly web-applications, adapted for custom-designed tNGS panels and dedicated to CNVs detection, have been recently developed to perform the analysis of SNVs, CNVs and indels in a single integrated workflow. In Figure 1 and Figure 2, we show examples of CNVs detection by using tNGS with two panels we have previously used to scan the coding regions of genes implicated in LSDs [5,129]. Deep sequencing data were analyzed with the user-friendly web-application Ion Reporter Software developed by Ion Torrent, which relies on normalized read coverage (DoC) across amplicons to predict also the copy number state, corrected for GC bias and compared with a baseline coverage constructed by the user from control samples with known ploidy.

Figure 1 shows two genomic losses in *MCOLN1* (panel a) and *CTNS* (panel b) detected in two DNA samples from girls affected by Mucolipidosis IV and nephropathic cystinosis, respectively. The first CNV (panel a) is a ~6 kb heterozygous deletion (ploidy state 1) spanning from exon 1 to exon 7 of *MCOLN1* gene (the same patient carried also a A>G transition in the acceptor splice site of intron 3 in the other allele, rs104886461); the second panel (b) shows a homozygous ~18 kb deletion (ploidy state 0) of *CTNS* encompassing exons 1–10, responsible for the pathology.

Figure 2 shows two further genomic losses involving *GALC* (panel a) and *GAA* (panel b) exons present in DNA samples from patients affected by Krabbe and Pompe diseases, respectively. Sequencing reads were generated by using a custom developed NGS panel targeting six LSDs-related genes, previously described in [5]. The first CNV (panel a) is a homozygous deletion of ~16 kb in *GALC* (ploidy state 0) resulting in the loss of exons 11–17, successfully identified by the Ion Reporter Software developed by Ion Torrent. The second CNV (panel b) consists of a small heterozygous single exon (exon 18) deletion of *GAA* (ploidy state 1), characterized by low confidence and low sensitivity values (these two parameters are sensible to CNV size). The same patient also carried a T>G transversion in an acceptor site in the second allele (rs38683423). Using the confidence and sensitivity values recommended by the Ion Reporter Software, the CNV shown in Figure 2, panel b, would be filtered out, and may determine its exclusion by an unconfident user. Therefore, it is particularly important to pay attention to applying stringent filters, and the analysis of previously characterized positive controls is strongly recommended in order to evaluate both performances and limits of targeted custom NGS panels and correlated bioinformatic analysis.

Along with the technical criticisms related to adequate SVs calling and visualization, the interpretation of their clinical relevance still remains an open current challenge, especially for CNVs. Indeed, the speed at which novel genetic variants are identified by NGS applications is far greater than our ability to interpret data and assign them pathogenicity significance. Therefore, determining what a non-recurrent or a de novo SV actually means for an individual’s health and what is its clinical meaning remains a very difficult task, which commonly generates discrepancies in inter-laboratory practices [130].

Although there are no generally established rules for analysis, interpretation and classification of VUS (Variants of Uncertain Significance) and de novo SVs, some attention should be emphasized before reporting a SV in a diagnostic report. For example, it is important to correlate the identified SV with the downstream metabolic activity, to assess the impact of the variant in multiple mRNAs transcripts and protein generated, to verify family segregation in order to understand if the family history of the disease is consistent with de novo inheritance, to investigate the *cis* or *trans* position of the SV compared to a second mutation. It is fundamental to query dedicated public databases (GnomAD, DGV, ClinVar, Decipher) and verify if the identified variant overlaps with other items already described (pathogenic, likely pathogenic, benign, likely benign), to evaluate the frequency in local population and to use a proper prediction tool for SV interpretation (e.g., SV interpreter) [131,132].

It should be kept in mind that, when targeted NGS panels are used, it is not possible to resolve the precise SV configuration and breakpoints; therefore, it could be necessary to use other molecular approaches, such as aCGH, long-read genome sequencing and gene expression analysis to investigate pathogenicity and characterize the possible mechanisms of complex SVs formation [133]. Moreover, given the constantly evolving landscape of SVs, it could be useful to include, in both the policies of the lab and in the informed consent signed by patients, the possibility of re-interpreting and updating the VUS and recontact patients [134].

Another important point concerns the emergence of newborn screening (NBS) programs for LSDs, which are taking place in the form of pilot studies or regional/national initiatives worldwide, and are mainly based on biochemical assays evaluating enzymatic activity or substrates accumulation (i.e., digital microfluidic fluorometry and tandem mass spectrometry) [2,5]. In the era of NBS programs, the search for confirmatory diagnosis on an asymptomatic neonate with a positive finding from a biochemical enzyme test is a great challenge and genotyping is becoming a primary tool, in which even the analysis of SVs should not be underestimated.

## 5. Conclusions

Conventional diagnostic procedures are able to identify and diagnose most individuals with LSDs. However, a number of cases remain unexplained for difficulties in detecting CNVs or other complex rearrangements altering gene expression and enzymatic function. Numerous case-reports have highlighted the advantages obtained by the use of NGS for the diagnostic validation of LSDs, especially for the detection of SVs in cases for which conventional procedures determine insufficient evidence. The clinical interpretation of complex non-recurrent SVs is still limited and sharing phenotypic and molecular information about patients is essential to define SVs’ clinical relevance and impact on the disease. Additional efforts are ongoing and should be implemented by the scientific community to standardize NGS procedures and data evaluation for introducing this type of analysis in clinical diagnostics of LSDs.

## Data Availability

Not applicable.

## Supplementary Materials

The following supporting information can be downloaded at: https://www.mdpi.com/article/10.3390/biomedicines10081836/s1, Table S1: Structural variants altering LSDs-related genes. References [135,136,137,138,139,140,141,142,143,144,145,146,147,148,149,150,151,152,153,154,155,156,157,158,159,160,161] are cited in the supplementary materials.
