# Supplementary material for: Detection of Structural Variants by NGS: Revealing Missing Alleles in Lysosomal Storage Diseases
Source: Biomedicines. 2022 Jul 29;10(8):1836. doi: 10.3390/biomedicines10081836 (PMC9405548; doi:10.3390/biomedicines10081836)
Supplement: Supplementary file 1 [file biomedicines-10-01836-s001.zip › biomedicines-1799454-supplementary.pdf]

Table S1. Structural variants altering LSDs-related genes.

CATEGORY	DISEASE	GENE	TYPE OF SV	PHENOTYPE MIM NUMBER	METHODOLOGY	REFERENCES
Lipid storage diseases	Farber disease	*ASAH1*	Deletion (g.8728_18197del (c.126-3941_382 + 1358del)	#228000	Long-range PCR and sequencing	[21]
	Metachromatic Leukodystrophy	*ARSA*	Whole gene deletion (14.5 kb)	#250100	Southern hybridization	[22]
	Krabbe disease	*GALC*	Deletion exons 12 and 14	#245200	qPCR and MLPA	[23]
			Exons 11–17 deletion		PCR	[135,136]
	Sandhoff disease	*HEXB*	Recurrent 5′-end 16 kb deletion	#268800	PCR and mRNA analysis	[24]
			5′-end 16 kb deletion and further partial deletions comprising intron 1-exon 2 and exons 1-5		PCR, MLPA and sequencing	[25]
			Deletion of the exons 1-5		MLPA	[26]
	Niemann-Pick disease, type A/B	*SMPD1*	Five deletions and three duplications (small and large)	#257200-#607616	PCR and sequencing	[27]
	Gaucher disease	*GBA*	Identification of a rare deletion (g.-3100_+834del3934) and 2 novel recombinant alleles (g.4356_7031conJ03060.1:g.2544_4568; g.1942_7319conJ03060.1:g.1092_4856)	#230800-#230900-#231000	MLPA	[28]
			RecΔ55, total gene deletion, RecNciI and Rec I		PCR and sequencing	[32]
			Complete gene deletion		Radioactively labelled PCR fragments	[137]
			Large deletions due to recombinant fusion allele, Allelic conversion and Recombinations in the 3' UTR region		qPCR	[138]

			Deletion of 3925 bp (overlapping 3091 bp of the 5'UTR, the first 2 exons and 351 bp of intron 2, due to an Alu–Alu recombination)		PCR, RT-PCR, Southern blot and sequencing	[31]
			Two recombinant alleles and one large deletion		Exome sequencing, MLPS and Long-range PCR	[10]
	Fabry disease	*GLA*	2914 bp deletion between introns 1 and 2	#301500	Nanopore sequencing	[39]
			Large deletion spanning across the exons 1 and 2, and exons 3 and 4		MLPA	[35]
			Putative interchromosomal recombination between two Alu elements of the GLA gene resulting in a large exonic duplication		Long-range PCR and NGS	[34]
	Wolman's disease	*LIPA*	Homozygous deletion c.(428 + 1_967-1)_(*1_?)del	#278000	qPCR	[40]
Mucopolysacchar idoses	MPS I	*IDUA*	Deletion of exon 14 and 3'UTR c.(1828 + 1_1829-1)_(*1963_?) del, and duplication extending from IDUA exon 2 to intron 12 c.(157 + 1_158-1)_(1727 + 1_1728-1)dup	#252800	MLPA	[42]
			Paternally inherited deletion of IDUA exons 1 and 2, c.(?_-88)_(299+1_300-1)del and a whole-gene deletion of IDUA (?_-88?)_(*136?)del secondary to maternal somatic mosaicism		PCR. MLPA and sequencing	[138]
	MPS IIIB or Sanfilippo type B	*NAGLU*	Alu-mediated deletion of exons 3-4 (1146 bp)	#252920	Long range and multiplex PCR	[45]
			Large deletion in exons 3–4		PCR-based library preparation and NGS	[46]
		HGSNAT	Heterozygous deletion of exon 15	#252930	NGS	[47]

	MPS IIIC or Sanfilippo type C		Deletion of exons 9-10		NGS	[43]
	MPS IIID or Sanfilippo disease type D	*GNS*	3 large deletions (Del EX1+, Del EX6,7 (c.625-637_875+6del3346ins8), delEX9-14 (c.1046_1659+16210del36529ins9)	#252940	PCR and mRNA analysis	[49]
			Intragenic deletion of 8723 bp encompassing exons 2 and 3		PCR and sequencing	[48]
	MPS IVA or Morquio A	*GALNS*	Alu-mediated deletions of exons 10–14 and exons 9–14	#612222	qPCR, mRNA analysis, aCGH, long-range PCR	[50]
			Double gene deletion: exons 2^5, exons 11-12		Reviewed in	[52]
			Large deletions fusing two genes: exons 3-14			
	MPS VI	*ARSB*	Deletion of exon 5	#253200	PCR and cDNA analysis	[56]
			Deletion of exon 4		qPCR	[55]
			Deletion of exons 2 and 3 (138 kb)		aCGH and sequencing	[54]
	Mucopolysaccharidosis type II, MPS II or Hunter Disease	*IDS*	Deletion of exon 3, Duplication of exon 5 to 9, Exon-1_6 Del, Deletion exon 1 and Duplication exon 4, Deletion of exon 4, Exon-8_9 Del, Whole Gene Deletion, Complex rearrangments	#309900	Reviewed in	[57]
			Large gene alterations (e.g. IDS/IDSP1 gene inversions, partial to extensive IDS deletions, and one chimeric IDS-IDSP1 allele)		PCR, cDNA analysis and aCGH	[58]
			Total IDS gene deletion; rearrangement involving a IDS gene inversion		qPCR and cDNA analysis	[59]
			Partial deletion that removes exons I to VI and extends about 200 kb upstream of the IDS gene; internal deletion of exons IV, V, VI, and VII		Southern blot, PCR and cDNA analysis	[139]

			5 Mb deletion Removing IDS and FMR-1		Karyotyping	[140]
			Deletion exons 2-4		PCR, sequencing and RNA analysis	[141]
			IDS/IDS2 inversion<br>Deletion of exons V, VI and VII Deletion of exons IV, V, VI, VII and VIII Deletion of exons V, VI and VII<br>Deletion of exons IV, V, VI, VII, VIII and IX		PCR, sequencing, aCGH	[41]
			9.4 Mb deletion; 3.9 Mb deletion of the Xq27.3–Xq28 and a 3.1 Mb duplication of the X q28 region; 41.8 Kb deletion		PCR and SNP array	[142]
			178 bp deletion in the promoter		PCR, cDNA analysis and sequencing	[72,143]
			3.2-megabase deletion encompassing *IDS*, FMR1, and *AFF2* (*FMR2*)		Microarray	[143]
			Deletion of exon 1 to exon 7		PCR, RFLP-PCR, and direct sequencing	[62]
			Several large deletions/complex rearrangements		PCR, sequencing, NGS	[71]
			Whole gene deletion		PCR	[144]
			Deletion of exon 7		MLPA, direct exon sequencing, and RFLP analysis	[64]

			Complete or partial gene deletion, and other rearrangments		Different molecular methodologies	[65,67,70,145-151]
			Exon 8 deletion		PCR and MLPA	[68]
			76 bp deletion		PCR, sequencing and cDNA	[152]
			Two deletions alternate with two duplications, overall affecting a region of about 1.2 Mb distally to IDS gene		aCGH	[69]
			43.6 kb deletion		Southern blot, PCR, DNA sequencing	[153]
			IDS/IDS-2 inversion		allele-specific PCR, MLPA and sequencing	[154]
			Recombinations IDS–IDS2, and large deletions		Sequencing, cDNA analysis and FISH	[66]
			Partial deletion in the long arm of chromosome X of paternal origin and a deletion of IDS inherited from the mother		PCR and direct sequencing analysis	[73]

Glycogen storage disease	Pompe disease	*GAA*	17kb deletion starting upstream of GAA in the CCDC40 gene and included the promoter, transcription start site (TSS), and non-coding exons 1A and 1B of GAA.	#232300	Splicing assay, minigene analysis, SNP array analysis, and targeted Sanger sequencin	[155]
			Exon 18 deletion (c.1293_1326+ 57del)		PCR and sequencing	[79]
			Exon 18 deletion (c.1293_1326+ 57del)		cDNA analysis and sequencing	[78,156]
			c.2481+102_2646+31del (delta exon 18)		PCR and sequencing	[79]
			Heterozygous 8-kb intragenic deletion (IVS7-19 to IVS15-17)		PCR	[77]
			Multiple exons deletion; exons 2-3 deletion		PCR and sequencing	[81]
			Exons 2–4 and exons 15–20 deletion		Whole exome sequencing	[80]
Glycoproteinoses	Fucosidosis	*FUCA1*	Two large deletion (encompassing exon 4, and exons 7/8) and a 66bp duplication in exon 6	#230000	Reviewed in	[85]
	Sialidosis type 1	*NEU1*	Deletion of exon 2	#256550	PCR and NGS	[86]
			Heterozygous 27.5 kb deletion involving the whole coding exons of NEU1		PCR and sequencing	[87]
	β-Mannosidosis	*MANBA*	Complex homozygous rearrangement characterized by a partial intragenic inverted duplication of MANBA, and resulted in the mRNA skipping of multiple contiguous exons	#248510	Whole-genome sequencing (WGS) and cDNA analysis	[88]
Disorders of post-translational modification	Mucolipidosis II alpha/beta	*GNPTAB*	Duplication of exon 2	#252500-#252600-#252605	PCR, cDNA analysisi and sequencing	[89]
			Alu–Alu-mediated large homozygous genomic deletion (897 bp) encompassing exon 19		Long-Range PCR and cDNA analysis	[90]
			Deletion of exon 9		qPCR and sequencing	[91]

Disorders of integral membrane proteins	Cystinosis	*CTNS*	57-kb deletion (including the first 10 exons)	#606272	Multiplex PCR, FISH	[92,93,157]
			13 kb deletion		Reviewed in	[102]
			266 bp duplication			
			Deletion of exons 4–5 (between 1.7 kb and 15 kb)			
			10 kb deletion			
			>1.7 kb deletion		MLPA	[102]
	Niemann-Pick disease, type C	*NPC1*	Whole NPC1 gene deletion	#257220- #607625	qPCR	[102]
			Large deletion including NPC1, C18orf8 and part of ANKRD29 gene; deletion encompassing the promoter region and exons 1–10 of NPC1 and the adjacent ANKRD29 and LAMA3		cDNA analysis, QMPSF and aCGH	[100]
		NPC2	Homozygous deletion of exons 2 and 3		Exome sequencing	[103]
	Mucolipidosis type IV	*MCOLN 1*	c.1_788deletion	#252650	Reviewed in	[104]
	Infantile free sialic acid storage disease	*SLC17A5*	Homozygous 94 bp deletion	#269920	NGS	[105]
			Homozygous deletion of exons 8-9		SNPs array, Sanger sequencing and aCGH	[106]
	Danon disease	*LAMP2*	Alu-mediated 34kb microdeletion encompassing the entire 5'UTR and exon-1; a 64kb and a 58Kb deletion ablating exons-4–10	°#300257	Genomic junction fragment PCR and and Southern Blot	[107]
			Alu-mediated Xq24 rearrangement causing a deletion encompassing *CUL4B*, *LAMP2*, and other three neighboring genes		PCR and karyotyping	[109]
			Multi exon-copy number variations		qPCR and NGS	[108]

			Alu-mediated tandem duplication of exons 4 and 5 (6.4 kb duplication)		PCR, cDNA analysisi and sequencing	[110]
Neuronal ceroid-lipofuscinoses	Neuronal ceroid lipofuscinosis, neuronal, type 3	*CLN3*	Common 1.02-kb intragenic deletion mutation (removing exons 7 and 8)	#204200	Different molecular methodologies	[84,112,158-161]
			Deletion exons 10-13			[159]
	Neuronal ceroid lipofuscinosis type 5	*CLN5*	Two Intragenic deletions encompassing exon 4	#256731	PCR and MLPA	[113]
			De novo large deletion at 13q21.33-q31.1		Exome sequencing	[114]
	Ceroid lipofuscinosis, neuronal, type 8	*CLN8*	De novo terminal deletion of the short arm of chromosome 8p23.3	#600143	FISH	[115]
			Deletion c.544-2566_590del2613 (2613 bp from intron 2 to exon 3)		PCR and sequencing	[116]
			Large deletions (400 kb, 54 kb and 235kb)		PCR, sequencing and aCGH	[117]
Disorders of LROs	Hermansky–Pudlak syndrome, type 1	*HPS1*	13.966-bp deletion	#203300	qPCR and sequencing	[119]
	Hermansky–Pudlak syndrome, type 2	*AP3B1*	8168 bp deletion including a big part of intron 14, the complete exon 15, and a small part of intron 15	#608233	Long-range PCR, cDNA analysis and sequencing	[121]
			Deletion of exon 14 and exons 10-25		PCR and mRNA analysis	[120]
			Pericentric inversion inv(5)(p15.1q14.1)		FISH and aCGH	[122]

References are listed at the end of the manuscript.
